# The complete chloroplast genome sequence of *Centella asiatica* (Linnaeus) Urban

**DOI:** 10.1080/23802359.2020.1768922

**Published:** 2020-05-22

**Authors:** Chan Li, Xuena Xie, Fang Li, Enwei Tian, Yuqi Shu, Zhi Chao

**Affiliations:** a Faculty of Medicinal Plants and Pharmacognosy, School of Traditional Chinese Medicine, Southern Medical University, Guangzhou, People’s Republic of China; b Department of Pharmacy, The Third Affiliated Hospital, Southern Medical University, Guangzhou, People’s Republic of China

**Keywords:** *Centella asiatica*, chloroplast genome, phylogenomic analysis

## Abstract

The apiaceous species *Centella asiatica* (Linnaeus) Urban is attractive not only to pharmaceutical researchers for its versatile medicinal uses, but also to botanists for its phylogenetic significance. We acquired its whole chloroplast genome (CP) through genome skimming. The CP genome of*Centella asiatica* was 154,771 bp in length, including a large single-copy (LSC) region with 86176 bp, a small single copy (SSC) region with 18107 bp, and a pair of inverted repeats (IR) regions with 25,343 bp. The whole AT content of the CP genome was 62.3%. Phylogenomic analysis revealed that *Centella asiatica* formed a separate clade sister to Saniculoideae and Apioideae species in the family Apiaceae. The work provides beneficial data for following researches on the genetic variation, species identification, phylogeny, and classification of *Centella.*

*Centella asiatica* (Linnaeus) Urban has been used medicinally to cure a number of maladies from prehistoric times. In China, the whole herb is used for its effectiveness in treating jaundice, heat stroke, fever, diarrhea, urinary stones, blood in urine, carbuncles and furuncles, and traumas, etc (Chinese Pharmacopoeia Commission 2015). Its effect on wound healing was described in World Health Organization monographs(World Health Organization 1999), and that on memory and cognitive ability enhancement was also attractive (Howes and Houghton 2003). The whole herb is also edible. People in the South China often drink it as herbal tea and also commonly take it as a food therapy material (Xiang et al. 2016). In Southeastern and South Asian countries, its fresh leaves can be used as vegetables or for juicing (Kosaka et al. 2013). Traditionally, *Centella* species were thought to be closely related to Hydrocotyloideae. Recent molecular phylogenetic studies led to the recognition that the Hydrocotyloideae was polyphyletic. In this study, we reported the CP genome of *Centella asiatica*, in order to inspect its systematic status from a phylogenomic perspective and to provide data base for developing molecular marker for this species.

The fresh leaves of *Centella asiatica* were obtained from the medicinal plant garden of Southern Medical University (113°19′43.35ʺE, 23°11′20.58ʺN), Guangzhou, China. Voucher specimen (voucher no. chaozhi2019042601) was identified by Professor Zhi Chao and was deposited in Southern Medical University herbarium (SMU, to be listed in the Index Herbariorum). Total genomic DNA was extracted from 100 mg fresh leaves using cetyltrimethyl ammonium bromide (CTAB) method (Yang et al. 2014). The pair-end sequencing was performed on Illumina HiSeq system at the Beijing Genomics Institution (BGI), in Shenzhen, China. The CP genome of *Centella asiatica* was assembled by SPAdes (Bankevich et al. 2012) with that of *Hydrocotyle sibthorpioides*as as a reference (Accession No. NC030260). The Geneious (v11.0.4) (Kearse et al. 2012) and Plastid Genome Annotator (PGA) (Qu et al. 2019) were used for genome annotation. The annotated sequence had been deposited in GenBank (Accession No. MN854377).

The complete CP of *Centella asiatica* displayed a typical quadripartite structure with 154,771 bp in length, including a large single-copy (LSC) region with 86,176 bp, a small single-copy (SSC) region with 18,107 bp, and a pair of inverted repeats (IR) regions with 25,343 bp. The complete CP including 113 genes, consisting of 79 protein-coding genes, 30 tRNA genes, and 4 rRNA genes. Among these genes, 15 genes had 1 intron while 3 genes had 2 introns. The over-all A-T content of the chloroplast genome was 62.30%.

A phylogenetic analysis was performed based on 21 CP genomes to reveal the phylogenetic position of *Centella asiatica*. The CP genomes were aligned with MAFFT (Katoh and Standley 2013) and then adjusted manually by Mega7 (Kumar et al. 2016). The maximum likelihood (ML) tree was inferred in IQ-tree based on GTR + R+I model using1000 bootstrap replicates. Phylogenomic analysis revealed that *Centella asiatica* formed a separate branch in the family of Apiaceae representing subfamily Mackinlayoideae, which is a sister group to the clades that included the species of Saniculoideae and Apioideae. Additionally, *Hydrocotyle* species formed a clade sister to the large clade of araliads, confirming its systematic position in Aralicaceae. The plastome based phylogeny was consistent with that proposed in the APG IV (Angiosperm Phylogeny Group 2016) (Figure 1).

**Figure 1.**
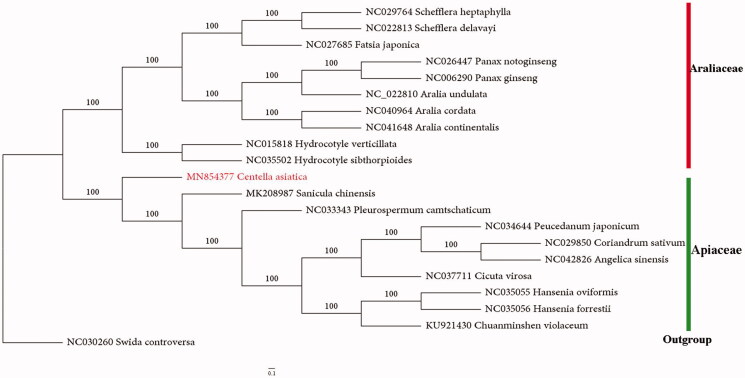
Maximum-likelihood (ML) tree based on the chloroplast genome of 21 taxa, including *Centella asiatica* and 1 outgroup taxon (*Swida controversa*). The bootstrap support values are indicated at the nodes.

## Supplementary Material

Supplemental Material

## Data Availability

The data that support the findings of this study are openly available in GenBank at https://www.ncbi.nlm.nih.gov/genbank/. See supplementary material for reference numbers.

## References

[CIT0001] Angiosperm Phylogeny Group. 2016. An update of the angiosperm phylogeny group classification for the orders and families of flowering plants: APG IV. Bot J Linn Soc. 181:1–20.

[CIT0002] Bankevich A, Nurk S, Antipov D, Gurevich AA, Dvorkin M, Kulikov AS, Lesin VM, Nikolenko SI, Pham S, Prjibelski AD, et al. 2012. SPAdes: a new genome assembly algorithm and its applications to single-cell sequencing. J Comput Biol. 19(5):455–477. 22506599 10.1089/cmb.2012.0021 PMC3342519

[CIT0003] Chinese Pharmacopoeia Commission. 2015. Pharmacopoeia of the People’s Republic of China, 2015 edition. Vol. 1. Beijing: China Medical Science andTechnology Press.

[CIT0005] Howes M-JR, Houghton PJ. 2003. Plants used in Chinese and Indian traditional medicine for improvement of memory and cognitive function. Pharmacol Biochem Behav. 75(3):513–527. 12895669 10.1016/s0091-3057(03)00128-x

[CIT0006] Katoh K, Standley DM. 2013. MAFFT multiple sequence alignment software version 7: improvements in performance and usability. Mol Biol Evol. 30(4):772–780. 23329690 10.1093/molbev/mst010 PMC3603318

[CIT0004] Kearse M, Moir R, Wilson A, Stones-Havas S, Cheung M, Sturrock S, Buxton S, Cooper A, Markowitz S, Duran C, et al. 2012. Geneious Basic: An integrated and extendable desktop software platform for the organization and analysis of sequence data. Bioinformatics. 28(12):1647–1649. 22543367 10.1093/bioinformatics/bts199 PMC3371832

[CIT0007] Kosaka Y, Xayvongsa L, Vilayphone A, Chanthavong H, Takeda S, Kato M. 2013. Wild edible herbs in paddy fields and their sale in a mixture in Houaphan province, the Lao People’s Democratic Republic. Econ Bot. 67(4):335–349.

[CIT0008] Kumar S, Stecher G, Tamura K. 2016. MEGA7: molecular evolutionary genetics analysis version 7.0 for bigger datasets. Mol Biol Evol. 33(7):1870–1874. 27004904 10.1093/molbev/msw054 PMC8210823

[CIT0009] Qu XJ, Moore MJ, Li DZ, Yi TS. 2019. PGA: a software package for rapid, accurate, and flexible batch annotation of plastomes. Plant Methods. 15(1):1–12. 31139240 10.1186/s13007-019-0435-7 PMC6528300

[CIT0010] World Health Organization. 1999. Herba Centellae. In: World Health Organization, editor, WHO monographs on selected medicinal plants. Vol. 1 (Original edition). Geneva: World Health Organization. pp. 77–85.

[CIT0011] Xiang JM, Xiao W, Xu LJ, Xiao PG. 2016. Research progress in *Centella asiatica*. Mod Chin Med. 18:233–238.

[CIT0012] Yang JB, Li DZ, Li HT. 2014. Highly effective sequencing whole chloroplast genomes of angiosperms by nine novel universal primer pairs. Mol Ecol Resour. 14(5):1024–1031. 24620934 10.1111/1755-0998.12251

