# Supplementary material for: The complete chloroplast genome sequence of Centella asiatica (Linnaeus) Urban
Source: Mitochondrial DNA B Resour. 2020 May 22;5(3):2149–50. doi: 10.1080/23802359.2020.1768922 (PMC7781932; doi:10.1080/23802359.2020.1768922)
Supplement: Supplemental Material [file TMDN_A_1768922_SM0255.docx]

**Table Accession numbers concerned in the article**

| **Serial Number** | **Species** | **Accession Number** |
| --- | --- | --- |
| 1 | *Centella asiatica* | MN854377 |
| 2 | *Sanicula chinensis* | MK208987 |
| 3 | *Pleurospermum camtschaticum* | NC033343 |
| 4 | *Peucedanum japonicum* | NC034644 |
| 5 | *Coriandrum sativum* | NC029850 |
| 6 | *Angelica sinensis* | NC042826 |
| 7 | *Cicuta virosa* | NC037711 |
| 8 | *Hansenia oviformis* | NC035055 |
| 9 | *Hansenia forrestii* | NC035056 |
| 10 | *Hydrocotyle verticillata* | NC015818 |
| 11 | *Hydrocotyle sibthorpioides* | NC035502 |
| 12 | *Chuanminshen violaceum* | KU921430 |
| 13 | *Schefflera heptaphylla* | NC029764 |
| 14 | *Schefflera delavayi* | NC022813 |
| 15 | *Fatsia japonica* | NC027685 |
| 16 | *Panax notoginseng* | NC026447 |
| 17 | *Panax ginseng* | NC006290 |
| 18 | *Aralia undulata* | NC022810 |
| 19 | *Aralia cordata* | NC040964 |
| 20 | *Aralia continentalis* | NC041648 |
